# Perceptions about the Implementation and Education of Telemedicine among University Students in Riyadh, Saudi Arabia

**DOI:** 10.12669/pjms.39.6.7797

**Published:** 2023

**Authors:** Shoukat Ali Arain, Juman Saleh Al Ajlan, Mohammed Ejaz Ahmed, Amjad Abdullah Alshehry, Sultan Ayoub Meo

**Affiliations:** 1 Shoukat Ali Arain Department of Pathology, College of Medicine, Alfaisal University, Riyadh, Saudi Arabia; 2 Juman Saleh Al Ajlan, College of Medicine, Alfaisal University, Riyadh, Saudi Arabia; 3 Mohammed Ejaz Ahmed, College of Medicine, Alfaisal University, Riyadh, Saudi Arabia; 4 Amjad Abdullah Alshehry, College of Medicine, Alfaisal University, Riyadh, Saudi Arabia; 5 Sultan Ayoub Meo Department of Physiology, College of Medicine, King Saud University, Riyadh, Saudi Arabia

**Keywords:** Telemedicine, Perceptions, Medical students, University students, Medical curricula

## Abstract

**Objectives:**

This study evaluated and compared the perceptions, awareness, and experiences of telemedicine among university students in Riyadh.

**Methods:**

In this cross-sectional study, a questionnaire was distributed electronically to undergraduate university students in Riyadh. The study was conducted from September 2021 to March 2022. Five-point Likert scale data were reported as percent agreement, while open-text comments were reported as recurring themes. Besides, the perceptions of medical students were compared with students from other disciplines.

**Results:**

Of 564 participants, 209 (37%) were medical students. Most respondents agreed that telemedicine could save patients’ time (77.7%) and improve access to healthcare (73.4%). The agreement was low for the statements that doctors would effectively evaluate the clinical features (39.2%) and that patients would effectively communicate their illnesses (44.3%). The agreement of medical students compared to students from other disciplines was even lower for these statements (25% vs. 47%; p<0.001) and (37% vs. 48%; p=0.03), respectively. Most medical students reported that they never learned about telemedicine (65%) and its tools (69%). Lack of awareness, training of healthcare workers and perceived lower quality of healthcare emerged as the most relevant factors for the limited acceptance of telemedicine.

**Conclusions:**

Most participants perceived that telemedicine could save patients’ time and improve access to healthcare. The low agreement, especially of medical students, for the ability of physicians to evaluate clinical features and of patients to communicate illnesses effectively possibly represented suboptimal education of telemedicine in medical curricula. Thus, incorporating telemedicine into medical curricula and improving public awareness might expedite telemedicine implementation.

## INTRODUCTION

Telemedicine and telehealth are often used interchangeably, with considerable overlap in scope. The use of telemedicine can be as simple as two doctors’ video conferencing about a case or as complex as a doctor performing robotic surgery remotely on an isolated patient.^1^ The World Health Organization defines telemedicine as, “the provision of healthcare services at a distance with communication conducted between healthcare providers seeking clinical guidance and support from other healthcare providers (provider-to-provider telemedicine) or conducted between remote healthcare users seeking health services and healthcare providers (client-to-provider telemedicine)”.^2^

Telemedicine may transform the future of medicine and overcome the many obstacles experienced by healthcare providers and their patients. For instance, one benefit is the expansion of healthcare services to patients in remote areas and the ability to reach a larger number of patients. Thus, it has the potential to improve healthcare quality, especially in rural settings. In addition to other valuable elements, its use in the management of chronic diseases is of great significance. Telemedicine offers various advantages over hospital visits, for example, in combating medication non-compliance; health literacy for the entire family can be promoted using phone or video calls. Telemedicine has been demonstrated to improve hypertension, diabetes, and rheumatoid arthritis management.^3^ Moreover, telemedicine is ideally suited to meet the demands of a high patient influx in COVID-like pandemics, simultaneously reducing virus transmission.^4^

The widespread availability of high-speed internet and the increasing usage of information technologies in traditional care have tremendously advanced the availability of remote health care.^5^ With electronic devices, such as mobiles, telephones, and laptops becoming a necessity, the growth and development of telemedicine are inevitable.^5^ Additionally, the COVID-19 pandemic has underscored the need for the utilization of technology in the form of telemedicine to protect both clinicians and patients.^6^-^8^

Despite its potential and tangible benefits, telemedicine adoption has not reached its maximum potential to benefit those in need.^9^ Certain technological, organizational, regulatory, and cultural challenges prevent telemedicine from being fully used. Scott et al.^10^ identified technical difficulties faced by staff as the most common barrier. Other common barriers were resistance to change from physicians and patients, the high cost of equipment, reimbursement issues, age > 40 years, and less than university-level education of patients.^10^ In Middle Eastern countries, it has been related to a lack of infrastructure and awareness, resistance to change, and lack of training in the use of information technology.^11^

The most significant factor interfering with the adoption of telemedicine is the lack of understanding of its acceptance among potential users. Recently, the perception of the viability of telemedicine in providing effective healthcare has improved. More than 90% of specialists in the four tertiary care hospitals in Riyadh agreed that telemedicine could save time and money.^12^ In addition, family physicians who used virtual services during the COVID-19 pandemic were generally satisfied.^13^ Obstacles in adopting telemedicine include patient privacy, high costs, lack of training, and communication between different stakeholders.^12^

Patient satisfaction is equally important for the successful implementation of any healthcare provision method. In the post-COVID-19 era, it appears that most patients are ready to adapt to telemedicine, especially for monitoring chronic diseases such as diabetes mellitus. The common advantages perceived by patients were convenience, timesaving, and access to a healthcare provider. Technical difficulties and a lack of physical examination were the most perceived disadvantages. In addition, low satisfaction was associated with an age group of more than 40 years and an education of less than the university level.^14^-^16^.

The incorporation of telemedicine into the Saudi healthcare system has increased since the start of the COVID-19 pandemic. However, this increase has not translated into a nationwide application of the technology. Emerging medical and university graduates from other disciplines will be potential frontline users of telemedicine in the near future. This study aimed to evaluate and compare perceptions, awareness, and experiences of telemedicine among university students in Riyadh, Saudi Arabia. In addition, we assessed the extent of knowledge gaps and missed opportunities for training medical students as well as the possible place of telemedicine training in medical curricula.

## METHODS

In this cross-sectional study, the data-gathering tool was a questionnaire designed in Google Forms by the investigators, using the existing literature. The questionnaire was validated and distributed electronically using e-mail addresses and WhatsApp groups to undergraduate students at various universities in Riyadh, Saudi Arabia. The study was conducted from September 2021 to March 2022.

The questionnaire was designed mainly on a five-point Likert scale, with a component having a list of factors to choose from and a component for open-text comments. It consisted of various sections, including demographic information, awareness of accessibility and convenience, quality of care, the relevance of ethics, and privacy to telemedicine services. In addition, participants had to choose the three most likely factors that, in their opinion, were the major contributors to the limited acceptance of telemedicine services out of a list of the seven most relevant reasons and were also asked to provide their suggestions for improvement. One component was included only for medical students regarding training in the use of telemedicine services.

### Ethical Approval

All data were collected anonymously. Participants were informed about the aim of the study at the beginning of the survey, and filling out the survey was considered as providing consent. Ethical approval for this study was obtained from the Institutional Review Board of Al Faisal University (Ref # IRB-20055).

### Statistical analysis

A five-point Likert scale (strongly disagree = 1; strongly agree = 5) was used for most of the questions in the survey. The Likert scale responses of “agree” and “strongly agree” were grouped as “agree”. Similarly, “disagree” and “strongly disagree” were lumped as “disagree”. The data presented in this study were analyzed using the Statistical Package for the Social Sciences version-27 (SPSS Inc., Chicago, IL, USA). Frequencies and percentages were calculated for all the nominal variables. Per cent, agreements for different survey items were calculated, and comparisons of the survey items were made using the chi-square test. A p-value of <0.05 was considered to show a significant difference in all the analyses. The open-text comments were independently reviewed by two authors (AJS and SAA) to identify recurring comments. Similar comments were coded as themes. Subsequently, the themes were compared to reach a consensus.

## RESULTS

A total of 564 students participated in the study. Most participants were females (80%) with ages ranging from 17 to 43 years, with a median age of 21 years (Table-I). In addition, the majority of the respondents were Saudi nationals (68%) living in cities (91%). Thirty-seven per cent of respondents were medical students.

**Table-I:** Demographic data of the study participants (N=564).

Variable	N (%)
***Gender***	
Male	110 (20)
Female	454 (80)
Median Age (in years)	21
***Ethnicity***	
Saudi	381 (68)
Arab (non-Saudi)	130 (23)
Non-Arab	53 (09)
***Hometown***	
City	512 (91)
Rural area/town	52 (09)
***Year of Study***	
First	147 (26)
Second	82 (15)
Third	126 (22)
Fourth	72 (13)
Fifth	78 (14)
Sixth	59 (10)
***Academic Major***	
Medicine	209 (37)
Allied Medical Sciences	58 (10)
English language & literature	112 (20)
Engineering/Computer science	96 (17)
Business	74 (13)
Others	15 (03)

Data are reported as frequency (%). Percentages are rounded-off to whole numbers.

Respondents’ overall perception of telemedicine use was favorable (Table-II). The strongest agreement was with statements that telemedicine could save patients’ time (78%) and improve access to healthcare services (73%). Conversely, the agreement was low for the statements that doctors would be able to evaluate the clinical features of patients effectively (39%), family/friends would prefer telemedicine over hospital visits for chronic illnesses (42%), and patients would be able to communicate their illnesses effectively (44.3%). In addition, 58% agreed that patients may have privacy concerns regarding their health information, while 51% agreed that patients may have concerns for cultural reasons. Participants were asked to rate their experience of telemedicine services, if any, on a scale of 1-10 (10 = excellent). Of the 211 respondents who responded to this question, 169 (80%) rated their experience as six or more.

**Table-II:** Perception of the participants on various aspects of telemedicine (N=564).

Survey Item	Agree	Neutral	Disagree
The use of telemedicine can save patients time	438 (78)	93 (16)	33 (6)
Access to healthcare services can be improved using telemedicine	414 (73)	115 (20)	35 (6)
Overall, it would facilitate doctor-patient interaction	357 (63)	150 (27)	57 (10)
For using telemedicine, reliable internet and telecommunication technologies are available	352 (63)	176 (31)	36 (6)
The use of telemedicine would reduce healthcare costs	345 (61)	158 (28)	61 (11)
Overall, telemedicine can provide quality medical care for chronic illnesses	338 (60)	159 (28)	67 (12)
Doctors will be able to communicate treatment plans and preventive instructions effectively	334 (59)	167 (23)	63 (11)
Patients may have privacy concerns about their health information being exchanged electronically	327 (58)	160 (28)	77 (14)
Patients may have concerns due to cultural reasons	289 (51)	161 (29)	114 (20)
Patients will be able to communicate their illnesses effectively	250 (44)	206 (37)	108 (19)
If made available, family/friends would prefer telemedicine over hospital visits for chronic illnesses	239 (42)	190 (34)	135 (24)
Doctors will be able to evaluate the clinical features of their patients effectively	221 (39)	192 (34)	151 (27)

Data are reported as frequency (%). Percentages are rounded off to the whole number.

Perceptions of telemedicine were compared between medical students and students from other disciplines (Table-III). Medical students showed a significantly low agreement with the statements that patients will be able to communicate their illnesses effectively (37% vs. 48%; p = 0.03) and that doctors will be able to evaluate the clinical features of the patients effectively (25% vs. 47%; p < 0.001). There were no differences in the other statements related to telemedicine between the two groups.

**Table-III:** Comparison based on the major university subject of the participants.

Survey Item	Medicine (n=209)	Other majors (n=355)	p-value
The use of telemedicine can save patients’ time.	162 (78)	276 (78)	0.08
Access to health services can be improved using telemedicine.	152 (73)	262 (74)	0.10
Overall, it would facilitate doctor-patient interaction.	130 (62)	227 (64)	0.92
For using telemedicine, reliable internet and telecommunication technologies are available.	127 (61)	225 (63)	0.058
The use of telemedicine would reduce healthcare costs.	137 (66)	208 (59)	0.26
Doctors will be able to communicate treatment plans and preventive instructions effectively.	115 (55)	219 (62)	0.29
Patients may have privacy concerns about their health information being exchanged electronically.	125 (60)	202 (57)	0.45
Patients may have concerns due to cultural reasons.	115 (55)	174 (49)	0.37
Patients will be able to communicate their illnesses effectively.	78 (37)	172 (48)	0.03*
If made available, family and friends would prefer telemedicine over hospital visits for chronic illnesses.	78 (37)	161 (45)	0.10
Doctors will be able to evaluate the clinical features of their patients effectively.	53 (25)	168 (47)	<0.001*

* P-value indicates a significant difference; Data are reported as frequencies (%) of agreeing participants. Percentages are rounded off to the whole number.

One component of the questionnaire was directed only at medical students regarding their learning of telemedicine (Table-IV). The majority responded that they had never or rarely learned about telemedicine (65%) and were not familiar with telemedicine tools (69%). Sixty-seven per cent agreed that they wanted to acquire training. Thirty-seven per cent preferred its inclusion as part of professional skill courses, 30% favoured including it in hospital rotations, and 25% as a separate elective.

**Table-IV:** Education of telemedicine among medical students (N = 209).

Questions	Response categories	n (%)
How often do you learn about telemedicine in your university?	Never/ Rarely	136 (65)
	Sometimes	51 (24)
	Often	22 (11)
To what extent are you familiar with Telemedicine tools?	Not familiar	145 (69)
	Somewhat familiar	37 (18)
	Familiar	27 (13)
Would you like to acquire training in the use of telemedicine?	Disagree	33 (16)
	Neutral	36 (17)
	Agree	140 (67)
Telemedicine may be included in the curriculum as:	An Elective	52 (25)
	A part of professional skill courses	78 (37)
	A part of hospital training	63 (30)
	Not required at all	11 (5)
	Other	5 (2)

Data are reported as frequency (%). Percentages are rounded to whole numbers.

**Fig.1:**
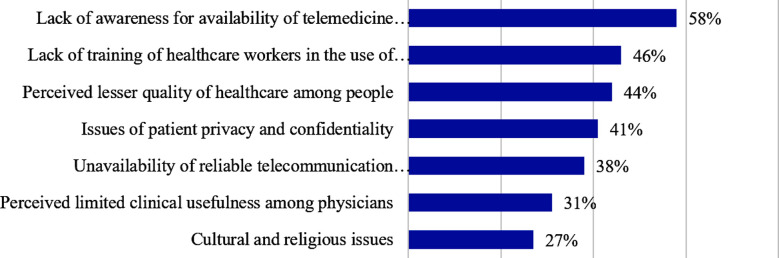
Most likely factors contributing to the limited acceptance of telemedicine services.

**Fig.2:**
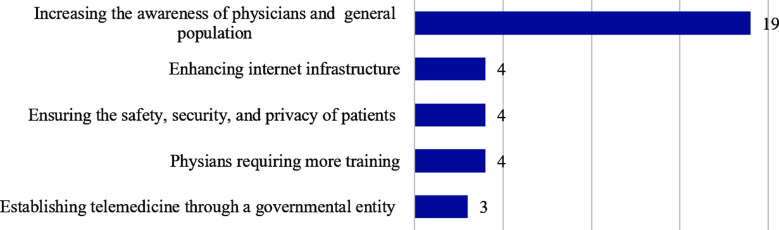
Common themes emerging from open-text comments to improve the use of telemedicine. (Numbers represent the number of comments under each theme).

Out of a list of possible factors, participants were asked to choose the three most relevant ones for the limited acceptance of telemedicine. Lack of awareness of the availability of telemedicine (58%), lack of training of healthcare workers (46%), and perceived lower quality of healthcare (44%) emerged as the three most common factors (Fig.1). Participants also suggested ways to improve the use of telemedicine in open-text comments (Fig.2). Increasing awareness of physicians and the public was suggested in 19 comments, which emerged as the most common theme.

## DISCUSSION

This study evaluated university students’ perceptions of various aspects of telemedicine, including its advantages and potential barriers. In addition, the perceptions of medical students regarding their knowledge of telemedicine were assessed. Among the 564 participating students, almost three-fourths agreed that the use of telemedicine could save patients’ time and improve access to healthcare services. Overall, less than half of the participants agreed with the statements that doctors would be able to evaluate clinical features and that patients would be able to communicate illnesses effectively. The agreement of medical students as compared to students from other disciplines was significantly low for these statements. Approximately two-thirds of medical students reported never learning about telemedicine. Lack of awareness and training of healthcare workers and perceived lower quality of healthcare emerged as the three most relevant factors for the limited acceptance of telemedicine.

The findings of this study are consistent with those of earlier studies reporting high satisfaction among patients and providers of virtual healthcare encounters in diabetes mellitus and across a spectrum of other diseases. In numerous studies, shorter travelling and waiting times have been identified as the most common advantages, resulting in timesaving, better accessibility to healthcare services, and ease of monitoring of chronic diseases.^15^-^17^.

This study showed low agreement for the ability of physicians to evaluate the clinical features of patients effectively and that of patients to communicate their illnesses effectively through telemedicine. In previous studies, opinions of healthcare providers have been diverse, based on the nature of the speciality and physicians’ willingness and capability to deliver healthcare and the quality of the available virtual healthcare delivery system. Besides, cultural, and ethical aspects also need consideration.^18^-^21^.

While compared with physicians, patients generally show tremendous satisfaction with the use of telemedicine, and these services have been proven effective in meeting the needs of different patient populations.^22^ Compared with medical students, university students of non-medical majors in our cohort perceived significantly better the ability of physicians to evaluate the clinical features and that of patients to communicate their illnesses effectively through telemedicine. On the part of medical students, this may reflect their lack of training in the use of telemedicine.

Similarly, approximately two-thirds of the medical students responded that they had not learned about telemedicine and were not familiar with telemedicine tools. In the open-text comments, one of the three most common factors for the limited use of telemedicine was identified as “lack of training of healthcare workers”. Educated and trained healthcare providers are likely to feel more comfortable delivering telemedicine.^23^-^25^.

Therefore, telemedicine learning is advocated for performing care procedures and overcoming the technical difficulties encountered during virtual sessions using telemedicine technologies.

### Limitations

Firstly, this study included only university students; therefore, the findings may not be generalizable to the overall perception of patients and providers regarding the use of telemedicine. Secondly, most of the participating students were from urban areas. Over half of the respondents (57%) belonged to Al Faisal University Riyadh. Rural populations may have different perceptions based on their awareness and availability of virtual technologies. However, our cohort represents the most relevant segment of the population in terms of service users and providers in the future. Additionally, the majority of participants did not have first-hand experience or exposure to telemedicine services. Therefore, their opinions may have been affected by a lack of knowledge about the use and recent advances in telemedicine technology.

## CONCLUSIONS

The overall perception of university students regarding the use of telemedicine was promising, especially for the benefits of saving patients’ time and improving access to healthcare services. The study showed low agreement for the ability of physicians to evaluate the clinical features of patients effectively and that of patients to communicate their illnesses effectively through telemedicine. Compared to non-medical university students, medical students had significantly lower agreeableness regarding the effectiveness of clinical evaluation and patient communication, possibly because of their suboptimal knowledge of telemedicine practices and tools. Consistent with these findings, lack of awareness of the availability of telemedicine, lack of training of healthcare workers, and perceived lower quality of healthcare emerged as the three most relevant factors for the limited acceptance of telemedicine. Increasing awareness among physicians and the public has emerged as the most common theme for improving telemedicine use. Thus, incorporating telemedicine into medical curricula and expediting public awareness is recommended to improve its acceptance by the medical fraternity and the population at large.

### Author Contributions:

**SAA**, **JSA** and **SAM:** Contributed to the design and manuscript writing.

**AAA** and **MEA:** Literature review.

**MEA**, and **JSA:** Data collection and analysis.

**SAM:** Responsible for the integrity and accuracy of this manuscript.

All authors have read and approved the manuscript.
